# Copaiba Oleoresin Improves Weight Gain and IL-10 Concentration, with No Impact on Hepatic Histology, in Liver Cirrhosis

**DOI:** 10.3390/biology13110853

**Published:** 2024-10-23

**Authors:** Maiara Taffarel, Bianca Sulzbacher da Silva, Angélica Macedo Borgês Paulino, Luciana Ortega Telles, Sabrina Trigueiro Mendonça, Cintia Vieira dos Santos, Morenna Alana Giordani, André Ferreira Nascimento, Danilo Henrique Aguiar, Valéria Dornelles Gindri Sinhorin, Carla Regina Andrighetti, Renata de Azevedo Melo Luvizotto, Gisele Facholi Bomfim

**Affiliations:** 1 NUPADS—Center for Research and Teaching Support in Health, Institute of Health Sciences, Federal University of Mato Grosso, Sinop 78550-728, MT, Brazil; maiara.taffarel.9@gmail.com (M.T.); biancasulzbacher@outlook.com (B.S.d.S.); borges.angelica94@gmail.com (A.M.B.P.); luciana.o.telles@hotmail.com (L.O.T.); sa_trigueiro@hotmail.com (S.T.M.); cintia_vieira.s@hotmail.com (C.V.d.S.); giordanimorenna@gmail.com (M.A.G.); nascimentoaf@yahoo.com.br (A.F.N.); reluvizotto@yahoo.com (R.d.A.M.L.); 2 ICS—Institute of Health Sciences, Federal University of Mato Grosso, Sinop 78550-728, MT, Brazil; crandrei20@yahoo.com.br; 3 ICNHS—Institute of Natural, Human and Social Sciences, Federal University of Mato Grosso, Sinop 78550-728, MT, Brazil; dha.danilo@gmail.com; 4 Postgraduate Program in Biotechnology and Biodiversity of the Pro Centro-Oeste Network, Federal University of Mato Grosso, Sinop 78550-728, MT, Brazil; valeria.sinhorin@ufmt.br

**Keywords:** copaiba oleoresin, liver cirrhosis, IL-10, thioacetamide

## Abstract

Copaifera sp. is a native tree in the Amazon region. Copaiba oleoresin has components such as sesquiterpenes, which have anti-inflammatory and antioxidant potential. Liver cirrhosis is the end stage of liver disease with limited therapeutic options. We aimed to evaluate the effect of copaiba oleoresin supplementation on the liver of animals with thioacetamide (TAA)-induced cirrhosis. For the induction of liver cirrhosis, 100 mg/kg of TAA was administered intraperitoneally twice a week for 8 weeks. A total of 200 mg/kg/day of copaiba oleoresin was administered via gavage for the same period. Copaiba oleoresin supplementation improved cirrhosis-associated cachexia by increasing weight gain and body fat. In addition, copaiba oleoresin attenuated systemic inflammation, as shown by the decrease in the circulating C-reactive protein. In the liver, the copaiba oleoresin decreased carbonyl proteins and increased IL-10 compared with TAA-treated rats. TAA groups demonstrated increased SOD, catalase, GST, and GSH activity in the liver. In conclusion, the supplementation of copaiba oleoresin demonstrated a beneficial systemic effect in alleviating cirrhotic cachexia and antioxidant and anti-inflammatory action in the liver. However, it failed to improve the serological and histological markers of liver damage, which could be associated with the advanced stage of the disease.

## Simple Summary

This study explores the potential benefits of copaiba oleoresin, a substance from a tree native to the Amazon, for treating liver cirrhosis, a severe and advanced liver condition with few treatment options. An animal model where cirrhosis was induced with a chemical called TAA was used. The animals were then given daily doses of copaiba oleoresin. The study found that the treatment helped the animals gain weight and body fat, which is often lost in cirrhosis, and reduced signs of inflammation. In particular, the liver showed higher levels of IL-10, a protein that helps reduce inflammation. Despite these benefits, copaiba oleoresin did not improve the overall liver damage markers, likely due to the severity of the disease. This suggests that while copaiba oleoresin may help manage some symptoms of cirrhosis, it may not fully reverse the condition at more advanced stages.

## 1. Introduction

Copaiba oleoresin, extracted from native Amazonian trees belonging to the Copaifera genus, is produced to detoxify the plant organism and defend the plant against animals, fungi, and bacteria [1]. Copaiba oleoresin contains a non-volatile resinous portion (diterpenes) and a volatile fraction composed mainly of sesquiterpenes, β-caryophyllene, α-humulene, α- and β-selinene, α-copaene, trans-α-bergamotene, and β-bisabolene [2,3]. In folk medicine, there are reports of using this oleoresin for its anti-inflammatory action and wound-healing properties [4,5,6]. These actions seem to be mediated by the most abundant sesquiterpenes components of this oleoresin, β-caryophyllene, and β-bisabolene, and also by the synergistic action of all components of the product [3,5,7,8,9,10].

Cirrhosis results from chronic inflammation in the liver with the involvement of increased oxidative stress [11,12]. Given this, copaiba oleoresin’s anti-inflammatory, antioxidant, and wound-healing properties make this natural product a potential approach to ameliorating cirrhosis.

Cirrhosis has a high degree of morbidity and mortality. Due to its limited treatment options, complications are responsible for more than one million deaths per year, and it is ranked as the 11th leading cause of death in the world [13,14]. According to the guidelines published by the European Association for the Study of the Liver and The Japanese Society of Hepatology and Gastroenterology, there is no specific treatment based on scientific evidence for liver fibrosis [15,16]. Therefore, it is crucial to investigate new compounds that can decrease the progression of liver cirrhosis.

At this stage of liver injury, there is a continuous loss of hepatocytes coupled with the production of reactive oxygen species (ROS) and fibrogenic mediators by cells of the innate immune system. Furthermore, there is an increase in the release of pro-inflammatory cytokines, such as TNF-α and IL-6, as well as a decrease in the expression of anti-inflammatory cytokines, such as IL-10 [12,17]. These mediators activate stellate cells that deposit collagen to heal the injured tissue, thus deforming the parenchyma and altering the vascular architecture, which leads to systemic changes [18,19,20]. Initially, the liver lesions are asymptomatic; however, as the disease progresses and becomes decompensated, liver dysfunction and portal hypertension lead to systemic manifestations, such as cachexia, weakness, jaundice, ascites, and others, in addition to an increased risk of hepatocellular carcinoma and death [18,21,22,23].

IL-10 has antifibrotic effects, as demonstrated by its capacity to suppress fibrogenic and pro-inflammatory gene expression [24,25]. A study demonstrated that IL-10 gene treatment attenuates liver fibrosis by inhibiting the activation and inducing the senescence of hepatic stellate cells and promoting the degeneration of collagen [25,26].

Therefore, considering the lack of treatment for liver cirrhosis and the anti-inflammatory and healing properties already described by copaiba oil–resin [27], we hypothesize that treatment with copaiba oil–resin improves the symptoms of cachexia and liver impairment present in thioacetamide-induced cirrhosis.

## 2. Materials and Methods

### 2.1. Animals and Experimental Model

Male Wistar rats, weighing approximately 200 g and 5 weeks old, from the Central Animal Facility of the Federal University of Mato Grosso (UFMT) on the Cuiabá Campus were used. Each group of rats was kept in collective cages in a light–dark cycle environment (12 h intervals), with a controlled temperature (24 ± 2 °C) and humidity (55 ± 5%). The study protocol followed the Ethical Principles in Animal Experimentation, recommended by the National Council for the Control of Animal Experimentation (CONCEA), and was approved by the UFMT Ethics Committee under number 23108.039273/2019-60. The animals were randomly divided into three groups: Control (C), Thioacetamide (TAA), and Thioacetamide + Copaiba Oleoresin (TAA + CO). Each group consisted of eight animals. All animals received water ad libitum and a standard specific diet for rodents containing carbohydrates (65.5%), proteins–casein (22%), lipids (4%), fibers (4%), vitamins (1%), mixture of minerals (3.5%), with a caloric value of 3.77 kcal/g, supplied by NUVILAB CR-1 (NuvitalVR, Colombo, Paraná, Brazil).

TAA (Sigma-Aldrich^®^, St. Louis, MO, USA) was diluted in saline and was administered intraperitoneally at a dose of 100 mg/kg in TAA and TAA + CO groups for 8 weeks twice weekly (on Monday and Thursday), based on previous studies from our group [19,21]. This drug has centrilobular hepatotoxic properties, and its metabolism generates an unstable and reactive metabolite that initiates oxidative damage and necrosis [28,29]. The oleoresin used was obtained from a commercial source and was analyzed by high-performance liquid chromatography, as described by Telles et al. (2022) [30]. Copaiba oil–resin was used in its native form and did not undergo any extraction method. The TAA + CO group also received copaiba oleoresin daily via gavage, diluted with 3% Tween 20 at a dose of 200 mg/kg for 8 weeks [31,32]. C and TAA groups were treated using treatment protocols to which they were not exposed.

At the end of the experimental period, the animals fasted for 8 h and were anesthetized with a mixture of ketamine (113 mg/kg) and xylazine (7.4 mg/kg) at a dose of 0.15 mL/100 g intraperitoneally. Blood samples (about 3 mL) were collected by cardiac punctures, and then the animals were euthanized by decapitation. The blood was centrifuged, and the serum was separated and stored at −80 °C for biochemical analysis. The liver was isolated, weighed, and stored for further analysis. Epididymal, retroperitoneal, and mesenteric fats were isolated and weighed. The calculation of weight gain was performed by subtracting the final body weight from the initial body weight during the treatment protocol.

### 2.2. Serum Biochemical Parameters

The concentrations of alanine aminotransferase (ALT), aspartate aminotransferase (AST), and C-reactive protein (CRP) were measured from serum. Blood samples were collected in Falcon tubes and centrifuged (3000 rpm; 10 min; Eppendorf^®^ Centrifuge 5804-R, Hamburg, Germany), and the serum was used for biochemical analysis using commercial kits (ALT and AST from Labtest—Lagoa Santa, MG—and CRP from Biotécnica Advanced—Varginha, MG).

### 2.3. Liver Histology

A part of the liver was fixed in 10% buffered formaldehyde for 24 h and washed with distilled water and then different ethanol concentrations. Subsequently, liver samples were dipped in pure resin and wetted with historesin. Cross sections of 3 µm were made with the microtome; then, the sections were washed, dried, and stained with hematoxylin and eosin (HE), placed in ovens, and clarified with xylol.

Histological sections of the liver were evaluated by an experienced investigator who was blinded to groups according to the published criteria for the analysis of the magnitude of inflammation and fibrosis. Fibrosis, inflammation, and hepatic steatosis levels were each assessed on a scale from 0 to 3 (0: not changed; 1: light; 2: moderate; 3: severe) [33,34,35,36].

### 2.4. Evaluation of Markers of Hepatic Oxidative Damage

In general, the liver was weighed, homogenized with the appropriate buffer for each technique at the appropriate dilution, and centrifuged, and the supernatant of the homogenates was used to measure oxidative damage markers.

Quantification of lipid peroxidation with thiobarbituric acid (TBARs) was performed by a spectrophotometry measurement at 535 nm using the malondialdehyde (MDA) curve as a standard and expressed as nmol MDA/mg protein. Protein carbonylation quantification was performed according to the method described by Colombo et al. (2016) by a microplate spectrophotometer measurement at 450 nm and expressed as nmol carbonyl/mg protein [37].

The activity of superoxide dismutase (SOD) was measured by spectrophotometry at 480 nm and expressed as UI SOD/mg protein [38]. Catalase activity was measured according to Nelson and Kiesow (1972) by spectrophotometry at 240 nm [39]. The difference in the absorbance reading at 60 s allowed for establishing the rate of H_2_O_2_ reduction, which was proportional to the rate of the enzymatic reaction catalyzed by catalase. The result was expressed as μmol H_2_O_2_/min/mg protein.

Glutathione S-transferase (GST) activity was determined according to Habig et al. (1974) and expressed as μmol GS-DNB/min/mg protein [40]. The non-enzymatic antioxidant, reduced glutathione (GSH), was measured according to Sedlak and Lindsay (1968) and expressed as μmol GSH/mg protein [41].

The determination of the proteins of all markers of hepatic oxidative damage, except for GSH, was performed by Bradford (1976) at 595 nm using bovine serum albumin as a standard [42].

### 2.5. Hepatic Cytokines

Liver samples weighing 50 mg were homogenized in phosphate-buffered saline (PBS). The homogenate obtained was centrifuged, and the supernatant was separated for cytokine measurements. IL-6, IL-10, and TNF-α were measured by ELISA using commercial kits (IL-6 and IL-10 from R&D System, TNF-α from Biolegend, San Diego, CA, USA). The concentration of each cytokine was calculated using a linear regression analysis of the standard curve obtained with rat recombinant cytokines.

### 2.6. Statistical Analysis

Data obtained were represented as the mean ± standard deviation. Data were submitted to the D’Agostino–Pearson normality test. One-way ANOVA was used to compare the groups, followed by Tukey’s post-test. For results that did not pass the normality test, the Kruskal–Wallis test, followed by Dunn’s post-test, was performed. The results were considered statistically significant for *p* values < 0.05. The graphs were generated, and the results were analyzed using the Graph Pad Prism 8.0 statistical program.

## 3. Results

Cirrhosis decreased the final body weight and epidydimal, retroperitoneal, and visceral fat compared with the control (Table 1). Copaiba oleoresin reversed all these altered parameters and also had a higher weight gain versus the TAA group, improving the general condition of the animals. There was hepatomegaly, observed by the increase in liver weight in the TAA group compared to the C group, and the TAA + CO group increased the liver weight even more. Liver damage occurred in the TAA and TAA + CO groups, as shown by an increase in ALT and AST concentrations, without an improvement with the administration of copaiba oleoresin. The inflammatory marker CRP increased in cirrhotic animals, and copaiba oleoresin treatment attenuated this parameter at values below the control group.

**Figure 1:**
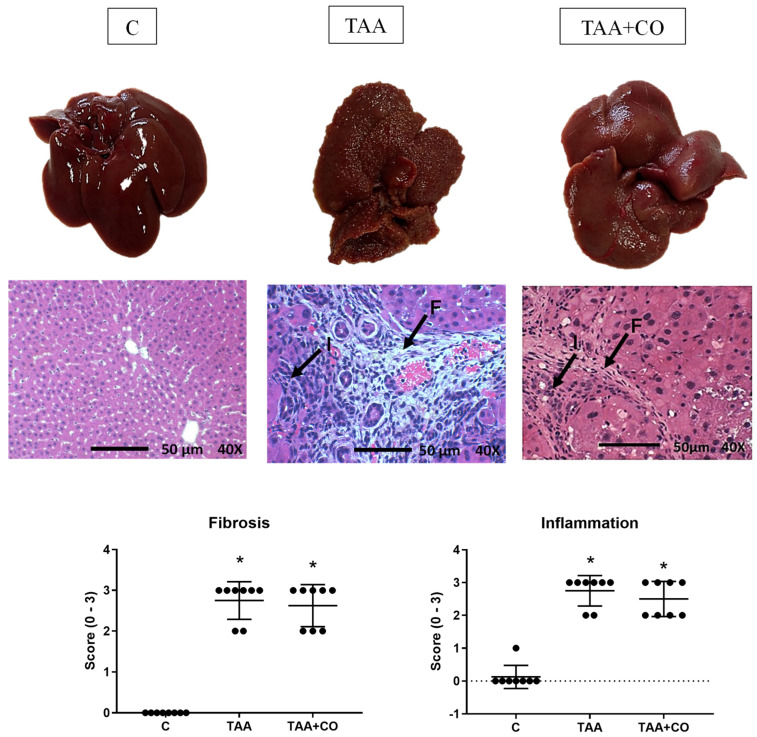
Macroscopic and microscopic appearance and histological analysis of the liver. Results are presented as mean ± standard deviation. * *p* ≤ 0.05 versus C; F: fibrosis; I: cellular infiltrates; C: control; TAA: thioacetamide; TAA + CO: thioacetamide and copaiba oleoresin. Fibrosis and inflammation levels were each assessed on a scale from 0 to 3 (0—not changed; 1—light; 2—moderate; 3—severe). The black arrows indicate histological features of inflammatory cell infiltrates (I) and fibrosis with collagen deposition (F). The oxidative marker analysis (Figure 2) demonstrated a significant decrease in protein carbonylation in the TAA + CO group compared with other groups. The TBARs, the enzymatic antioxidants, GST, SOD and catalase, and the non-enzymatic antioxidant GSH were increased in the TAA and TAA + CO groups when compared with the control.

The histological analysis revealed a higher score in fibrosis and inflammation (Figure 1) in the TAA and TAA + CO groups versus the C group, with no steatosis-related changes.

**Figure 2:**
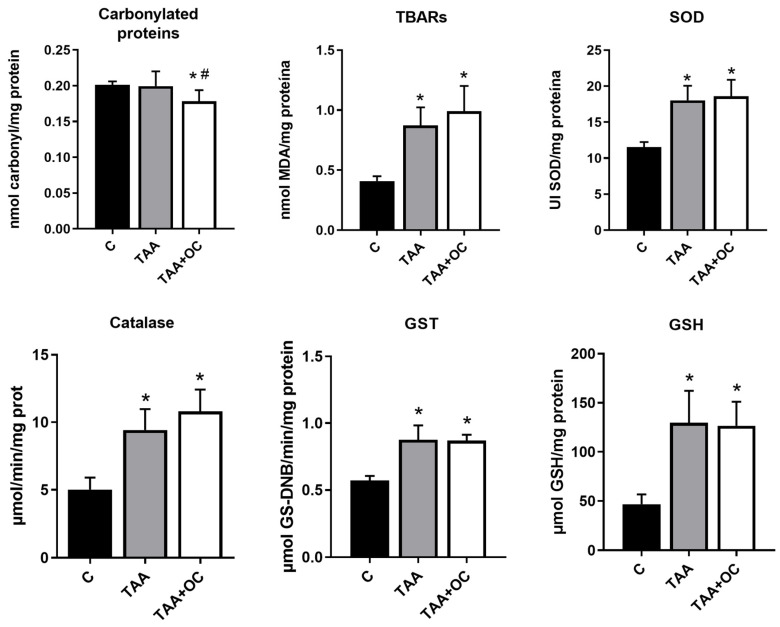
Parameters of liver oxidative damage in the control, cirrhosis, and copaiba oleoresin intervention animal groups. Results are presented as mean ± standard deviation. * *p* ≤ 0.05 versus C; # *p* ≤ 0.05 versus TAA. n: 8. C: control; TAA: thioacetamide; TAA + CO: thioacetamide and copaiba oleoresin; TBARs: thiobarbituric acid reactive substance; SOD: superoxide dismutase; GST: glutathione-S-transferase; GSH: reduced glutathione.

The oxidative marker analysis (Figure 2) demonstrated a significant decrease in protein carbonylation in the TAA + CO group compared with the other groups and an increase in TBARs in the TAA and TAA + CO groups compared with C. The enzymatic antioxidants, GST, SOD and catalase and the non-enzymatic antioxidant GSH were increased in the TAA and TAA + CO groups when compared with the control.

**Figure 3:**
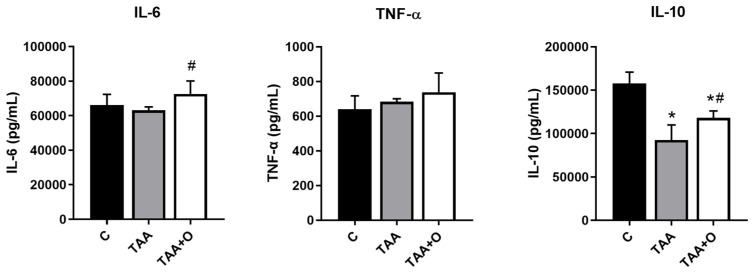
Liver cytokines of animals in the control, cirrhosis, and copaiba oleoresin intervention groups. Results are presented as mean ± standard deviation. * *p* ≤ 0.05 versus C; # *p* ≤ 0.05 versus TAA.. n: 8. C: control; TAA: thioacetamide; TAA + CO: thioacetamide and copaiba oleoresin; TNF-α: tumor necrosis factor alpha; IL-6: interleukin 6; IL-10: interleukin 10.

An increased IL-6 concentration was observed in the TAA + CO group compared with TAA (Figure 3), but there was no difference when compared with the control. There was no change in TNF-α levels between all groups. We observed an IL-10 reduction in the TAA group compared with C and TAA + CO. The copaiba oleoresin administration significantly increased the levels of IL-10 in the liver in cirrhotic animals when compared with the TAA group.

## 4. Discussion

The main findings of this study were that daily supplementation with copaiba oleoresin improved cirrhosis-associated cachexia by increasing weight gain and body fat while also reducing systemic inflammation, as evidenced by decreased circulating CRP levels and liver oxidative stress. However, no significant differences were observed in the liver histology or serological markers of liver injury (ALT and AST) with oleoresin treatment. In cirrhosis, liver damage impairs food intake and metabolism, contributing to cachexia, which was reflected in the reduced weight gain and fat mass in the TAA group [22,43,44]. The administration of copaiba oleoresin in cirrhotic animals restored visceral fat to control levels and significantly improved other health parameters, suggesting its role in the recovery of the animal’s overall condition.

Clinically, cirrhosis is diagnosed by symptoms, signs, and test results related to liver function. The serum markers most associated with liver damage are AST and ALT enzymes. These enzymes are expressed in the liver, contribute to gluconeogenesis, and are released into the circulation during liver injury [45]. Another gold standard observation for the liver cirrhosis diagnosis is the observation of the fibrosis and inflammation histological features characteristic of cirrhosis. In this experiment, supplementation with copaiba oleoresin did not decrease the high concentration of AST and ALT liver enzymes or the histological characteristics of inflammation and fibrosis present in animals with liver cirrhosis. Studies have not reached a conclusive finding on whether copaiba oleoresin treatment improves hepatic histology and ALT and AST levels in liver injury [46,47]. One of the reasons that may have led to this lack of improvement in the liver is the degree of liver damage of the experimental model used, since treatment for 8 weeks at a dose of 100 mg/mL of thioacetamide generated a grade-three inflammatory and histological lesion, causing great deformation of the hepatic tissue that would be difficult to be regenerated.

Another reason that may have led to no direct improvement in the histological and functional aspects of the liver is that the copaiba oleoresin used had a lower concentration of β-caryophilene, an important sesquiterpene with known anti-inflammatory, antioxidant, and healing effects. We also considered increasing the dose concentration. Even though our pilot experiment showed no toxicity at the initial dose, and we aimed to use a typical dose for individuals using copaiba oleoresin, we found that this dose did not provide significant benefits in cases of severe liver impairment. However, the sample used showed a greater number of other sesquiterpenes, such as trans-α-bergamotene, β-selinene, β-bisabolene, and α-copaene. β-bisabolene has anti-inflammatory, bactericidal, and insecticidal properties [1,6,47]. In a study using a sample of oleoresin with comparable amounts of these sesquiterpenes, the authors showed an anti-inflammatory activity corroborating with the results of CRP and IL-10 found in our analysis [7].

As for the molecular findings, the presence of oxidative stress in the liver of the cirrhotic groups was observed by the increased levels of TBARs, demonstrating that TAA induced oxidative damage in the liver, as previously shown [21,29]. Copaiba oleoresin demonstrated an antioxidant effect by decreasing carbonylated proteins in the liver of cirrhotic animals. This decrease had also been observed by Ghizoni et al. (2017) with an experimental model of rheumatoid arthritis [9]. Pereira et al. (2018), in their study, using extract from the stem bark of *Copaifera multijuga* in a model of paracetamol-induced liver injury, associated the observed antioxidant action to the presence of phenolic compounds in the extract, including flavonoids and tannins [48]. Nevertheless, an increase in the antioxidant activity was observed in cirrhotic animals. This could be an indication of a compensatory mechanism attempting to restore the redox imbalance present in cirrhotic animals [49].

CRP is a fundamental nonspecific serological marker of inflammation, and we observed an increase in CRP in the cirrhotic group, corroborating the ongoing inflammatory process [14,50], which was attenuated by copaiba oleoresin administration. The anti-inflammatory potential of copaiba oleoresin was previously demonstrated by Gomes et al. (2010), and Veiga et al. (2007) found the best activity in oleoresin from *Copaifera multijuga* Hayne [1,4]. Despite histological confirmation of inflammation, our molecular analysis of pro-inflammatory cytokines (IL-6, TNF-α) showed no increase in the liver of cirrhotic animals, likely due to the limited duration of the innate immune response (Abbas et al., 2012). This contrasts with Turkseven et al. (2020), who observed an increase in cytokine gene expression in a bile duct ligation model, which causes acute liver injury, unlike our chronic liver injury model induced by eight weeks of TAA administration.

IL-10 is an anti-inflammatory cytokine that inhibits the release of pro-inflammatory cytokines and the immune response activation [24]. IL-10 polymorphism has already been found to increase the risk of developing cirrhosis [51]. Therefore, one of the mechanisms that may be responsible for the greater inflammatory response in the liver of cirrhotic animals is the decrease in IL-10. In the CCl4 cirrhosis model, liver fibrosis attenuated in the presence of IL-10 [27]. Supplementation with copaiba oleoresin caused an increase in IL-10 concentration in the liver, suggesting an anti-inflammatory response stimulated by oleoresin. The higher concentration of IL-10 in the TAA + CO group may also be related to decreased oxidative stress, as this cytokine can inhibit the release of oxidative compounds [20].

A limitation of this study is that we did not isolate the compounds present in the copaiba oleoresin but instead used the oleoresin in its native form, similar to those consumed by people from the Amazon region. Therefore, it was not possible to discern whether the anti-inflammatory and antioxidant action occurred due to the individual action of any component or the synergistic effect of all components of the copaiba oleoresin used.

## 5. Conclusions

Copaiba oleoresin has an important systemic action, improving cirrhotic cachexia by increasing weight gain and fat weight as well as reducing systemic inflammatory markers. In the liver, there was antioxidant action and an increase in IL-10. There was no difference in the serological and histological markers of liver damage associated with liver cirrhosis after supplementation with copaiba oleoresin. This lack of difference could be due to a high-severity lesion in the liver or the dose used in this study.

## Figures and Tables

**Table 1:** Morphological and serological data of animals in the control group, with cirrhosis and with copaiba oleoresin intervention.

Parameters	Groups		
	C	TAA	TAA + CO
FBW (g)	436.0 ± 16.67	369.0 ± 21.03 *	396.2 ± 19.13 *^#^
Weight Gain (g)	136.3 ± 16.84	46.0 ± 13.64 *	68.8 ± 9.10 *^#^
Epididymal Fat (g)	9.3 ± 0.42	5.5 ± 0.55 *	6.5 ± 0.75 *^#^
Retroperitoneal Fat (g)	13.5 ± 1.35	7.6 ± 0.90 *	9.2 ± 1.01 *^#^
Visceral Fat (g)	5.8 ± 0.51	4.6 ± 0.47 *	6.0 ± 0.93 ^#^
Liver/BW (g)	26.5 ± 1.04	38.1 ± 2.06 *	40.8 ± 2.16 *^#^
ALT (U/L)	77.1 ± 9.54	151.1 ± 15.01 *	198.0 ± 67.05 *
AST (U/L)	263.6 ± 37.14	455.1 ± 53.43 *	400.8 ± 72.1 *
CRP (mg/dL)	2.5 ± 0.52	3.6 ± 0.55 *	1.8 ± 0.30 *^#^

Results are presented as mean ± standard deviation. * *p* ≤ 0.05 versus C; ^#^ *p* ≤ 0.05 versus TAA. n: 8. C: Control; TAA: thioacetamide; TAA + CO: thioacetamide and copaíba oleoresin; FBW: final body weight; BW: body weight; ALT: alanin aminotransferase; AST: aspartate aminotransferase; CRP: C-reactive protein.

## Data Availability

The data that support the findings of this study are available from the corresponding author upon reasonable request.
